# Validity of On-Line Supervised Fitness Tests in People with Low Back Pain

**DOI:** 10.3390/healthcare11071019

**Published:** 2023-04-03

**Authors:** Ana Myriam Lavín-Pérez, Juan Luis León-Llamas, Francisco José Salas Costilla, Daniel Collado-Mateo, Raúl López de las Heras, Pablo Gasque Celma, Santos Villafaina

**Affiliations:** 1 Centre for Sport Studies, Rey Juan Carlos University, 28943 Fuenlabrada, Spain; 2 GO fitLAB, Ingesport, 28003 Madrid, Spain; 3 Physical Activity and Quality of Life Research Group (AFYCAV), Facultad de Ciencias del Deporte, Universidad de Extremadura, 10003 Cáceres, Spain; 4 Sports Medicine Service, Alcobendas City Council, 28100 Alcobendas, Spain; 5 Department of Physical Education, Sport and Human Motricity, Autónoma Univesity, Ciudad Universitaria de Cantoblanco, 28049 Madrid, Spain; 6 Departamento de Desporto e Saúde, Escola de Saúde e Desenvolvimento Humano, Universidade de Évora, 7004-516 Évora, Portugal

**Keywords:** exercise, physical fitness, pain, patient outcome assessment, online intervention

## Abstract

This study aimed to investigate the concurrent validity between online evaluations (OEs) and face-to-face evaluations (IPEs) of a Senior Fitness Test and two balance tests in people with low back pain (LBP). Forty participants of 58.48 (9.87) years were included. The 30 s chair stand-up, arm curl, 2 min step, chair-sit and reach, back scratch, 8 foot up-and-go, sharpened Romberg, and one-legged stance tests were administrated using both OE and IPE methods. The results indicated no significant differences (*p* > 0.05) between the two methods except in the 8-foot up-and-go test (*p* = 0.007). Considering the ICC values and Bland-Altman plots, excellent agreement was found for the chair-sit and reach test, moderate agreement for the arm-curl and 8-foot up-and-go tests, and good agreement for the other tests. Strong correlations (*p* < 0.001) were observed in all variables except for the arm-curl and 8-foot up-and-go tests, where moderate correlations were found (*p* < 0.05). These results support the validity of OEs and IPEs in all tests, except for the arm-curl and 8-foot up-and-go tests, where lower ICC values and moderate correlations were found. However, it is important to consider the range of fluctuation of the ICC and the significant values obtained through correlations.

## 1. Introduction

Low back pain (LBP) is the main contributor to the overall burden of musculoskeletal conditions (568 million prevalent cases worldwide, responsible for 47% of global years of healthy life lost due to disability, YLDs), and neck pain is the fourth (223 million people; 22 million YLDs) [1]. Moreover, LBP is a global health problem [2]. Moreover, this is a global health problem [2] with a growing prevalence from 377.5 million people in 1990 to 577 million in 2017 and that entailed a health cost in the US of $56.5 and $62.3 thousand billion annually, respectively, an increase of 112% since 1990 [3].

LBP has a multifactorial and complex pathology that can cause significant limitations to the activities of daily living [4]. This condition normally produces a decline in the physical activity level, caused by kinesiophobia or pain-catastrophizing [5]. Consequently, sedentary behavior could affect patients’ physical fitness, reducing physical function and strength [6], which may worsen their prognosis. In addition, LBP prevalence increases with age, and it is related to a higher body mass index, less physical activity, and lower self-perceived health [7]. Thus, exercise could be a useful tool, as long as there is no medical contradiction and it is individualized, to prevent and rehabilitate LBP, improving patients’ quality of life and the ability to perform activities of daily living [8]. Accordingly, to design an individualized and effective physical exercise intervention, it is necessary to perform physical fitness tests [9]. In addition, to have as complete a picture as possible of the person’s clinical condition, it would be advisable to take into account parallel measures, such as the level of pain through specific assessments [10] or the risk of developing this LBP and the predictors of this condition [11].

Regarding physical fitness evaluations in people with LBP, the American College of Sports Medicine (ACSM) recommends the evaluation of cardiorespiratory fitness, strength, and flexibility [12]. The Senior Fitness Test Battery (SFT) [13] consists of six tests that assess the strength of lower and upper limbs (arm curl and chair stand tests), the flexibility of the upper and lower limbs (back scratch and chair sit and reach), cardiorespiratory fitness (the 6 min walking test or the 2 min step test can be used), and agility (8-foot up-and-go test). Thus, the Senior Fitness test battery (SFT) [13] is a good option to evaluate people with LBP. However, previous studies suggested that balance could also be evaluated in people with LBP due to an increased center of pressure sway compared with that in the healthy controls [14,15]. In this regard, previous studies have included complex and challenging balance tasks since they are more sensitive in detecting balance impairments in people with LBP [16,17]. Among these challenging balance tests, the tandem Romberg test and the one-legged stance test have been proposed as challenging for the LBP population [18].

Considering the current health situation due to COVID-19 [19] and the growth of telemedicine, taking advantage of the emergence of new technologies and internet access, physical fitness assessments have considered new ways of monitoring and controlling the participants´ functionality and physical fitness condition. Previous studies have started to address this need by assessing patients with multiple sclerosis [20], chronic respiratory disease [21], cardiac conditions [22], and cancer [23] and even assessments of veterans during the COVID-19 pandemic using remote physical fitness evaluations [24]. Among these articles, two were systematic reviews that analyzed the test used remotely [21,22]. However, Hwang, Fan, Bowe, Louis, Bertram, Morris, and Adsett [22] analyzed the most used physical fitness test remotely used in cardiac conditions (in this case the 6 min walking test) without analyzing the validity of the remote physical fitness test. In the same line, Holland, Malaguti, Hoffman, Lahham, Burge, Dowman, May, Bondarenko, Graco, and Tikellis [21], after performing a systematic review of articles that included at-home, remote, and face-to-face evaluations of people with pulmonary diseases, concluded that physical fitness tests are rarely conducted remotely.

Nevertheless, there are studies that have conducted remote assessments of physical fitness. Among these studies, Blair, Blair, Harding, Herman, Boyce, Demark-Wahnefried, Davis, Kinney, and Pankratz [23] participants received a toolkit and instructions for setting up the timed-up-and-go and the 30 s chair stand test. However, data are still not available since it is a protocol study. Differently, the study of Ogawa, Harris, Dufour, Morey, and Bean [24] determined the inter-rater reliability of three physical fitness tests performed via a telehealth visit (30 s arm curl test, 30 s chair stand test, 2 min step test) among community-dwelling older veterans. However, these tests were focused on mobility or strength, without addressing other relevant capacities for activities of daily living, such as flexibility, aerobic capacity, or balance. Furthermore, the online evaluations conducted in these previous studies have been employed by using isolated tests without a comparison and validation between online and face-to-face evaluations. In contrast, Winters-Stone, et al. [25] reported the validity of the chair stand test and the 4 m usual walk test, showing acceptable validity when remotely performed for older adults with cancer. In the same line, Hoenemeyer, et al. [26] reported the reliability and validity of the SFT (including the sit and reach test, the 30-chair stand test, the back scratch test, the 8-foot up-and-go test and go test, the timed 8 min walk test, and the 2 min step test) in cancer survivors and supportive partners. Conclusions highlighted that remote physical fitness assessments are reliable, valid, acceptable, and safe in these populations.

Thus, due to the lack of evidence regarding remote physical fitness assessments for one of the most prevalent conditions [1], such as LBP, as well as the need to compare remote vs. face-to-face physical fitness assessments, this study aimed to evaluate the validity of a novel online adaptation of the six functional tests included in the SFT (30 s chair stand-up test, arm curl test, 2 min step test, chair-sit and reach test, back scratch test, and 8-foot up-and-go test) and two balance tests (the sharpened Romberg test and the one-legged stance test). In this regard, the hypothesis was that the validity between the OE and IPE will range from a good to excellent level of agreement based on the results of a previous study [26].

## 2. Materials and Methods

### 2.1. Study Design and Participants

**Figure 1:**
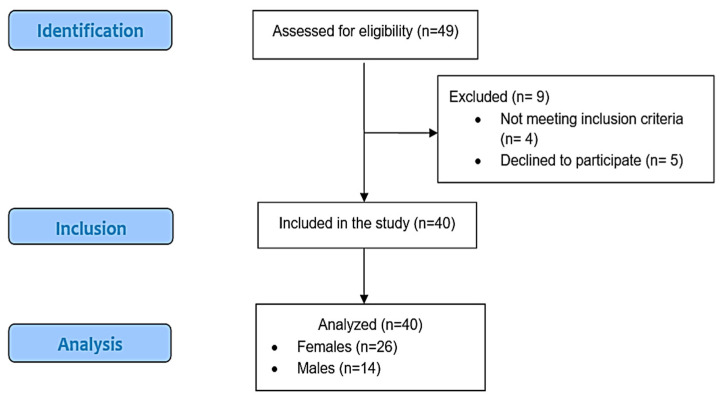
Flow diagram.

Considering the sample size calculation performed with the G*Power software 3.1.9.4 (Kiel University, Kiel, Germany), a minimum of 13 people was needed to achieve a 99% power to detect a significant correlation with an alpha of 0.001. For this purpose, regarding the different statistical tests presented in G*Power, the bivariate normal model was selected to correlate two variables analyzed in a former study and also included in the current study. Data of OEs and IPEs of the 8-foot up-and-go test provided by Hoenemeyer, Cole, Oster, Pekmezi, Pye, and Demark-Wahnefried [26] were used. Lastly, a convenience sample of 40 people (26 females and 14 males) with LBP were recruited in a Sport Medicine Service (Alcobendas, Spain) and enrolled in the study before June of 2021 (Figure 1). Since in this Sport Medicine Service, more than 13 people voluntarily decided to participate in this validity study, all of them were included for eligibility. Participants randomly performed a face-to-face (IPE) and an online evaluation (OE) in order to assess the validity of the SFT and two balance tests (sharpened Romberg test and one-legged stance test).

The specialist of the Sport Medicine Service recruited the patients from the LBP rehabilitation programs of the institution. In this regard, those patients who wanted to participate and fulfilled the following inclusion criteria were incorporated into the study: (a) people diagnosed with LBP, (b) people aged between 45 to 75-years-old, and (c) people without a medical contraindication for exercise. This age range was selected because it had the highest prevalence of low back pain in the sports medicine service of the city council. Moreover, the following exclusion criteria were considered: (a) people with other musculoskeletal or chronic diseases, (b) people without a phone, tablet, or computer with a camera and internet connection, (c) people injured or operated on in the last month, and (d) people with mental disorders unable to understand or execute physical indications. Concretely, the included participants had persistent pain beyond 3 months of symptoms [27] and one or more of the following diagnoses: herniated disc(s), scoliosis, spinal stenosis, and spinal osteoarthritis.

All the procedures were approved by the Research Ethics Committee of the University of Extremadura (approval reference: 41/2021) according to the ethical standards of the Declaration of Helsinki. The study was conducted from December 2020 to July 2021. Before starting the study, all patients were informed of the procedures and signed an informed consent form.

### 2.2. Procedures and Assessments

The validity of the SFT and two balance tests were carried out by randomizing the order of the evaluations according to the modality (face-to-face or online evaluations). For this process, a series of random numbers was generated using a randomization tool available on the web (https://www.randomizer.org/ accessed on 8 January 2022) that allowed the order of the evaluations to be established for each participant. Both evaluation sessions were developed under the supervision of a physical activity and sports science professional. Each kind of evaluation was performed by the same evaluator and in the same order starting with the non-fatiguing test, then the muscular endurance tests, and ending with the submaximal aerobic capacity test [28]. The two evaluation procedures were spaced by a 2-week period.

The SFT [13] has been chosen as it is widely used in clinical and research settings and has the ability to be performed easily and cheaply in homes or clinics without extensive technical expertise. The six physical fitness tests included in the SFT [13] were as follows: 30 s chair stand-up test, arm curl test, 2 min step test, chair-sit and reach test, back scratch test and 8-foot up-and-go test. Furthermore, since the balance evaluation of the challenging balance test is recommended for people with LBP, two validated balance tests (sharpened Romberg test and one-legged stance test) were included [29,30]. Two balance tests were evaluated since the one-legged stance test is more complex than the sharpened Romberg test. By introducing two levels of difficulty, more complete information about participants’ balance was obtained.

However, the arm curl test, chair-sit and reach test, and back scratch were adapted to be self-administrated (see Table 1). Before the evaluations, independently of the assessment modalities (OE or IPE), the professional explained to participants how to perform the tests. In this regard, the main differences between the types of evaluation were as follows: (1) the professional and participant setting, and (2) the way to collect participants’ results. In the IPE, the professional was next to the participants, with the material selected and placed, making the corresponding explanations and noting down the results that he measured. Whereas in the OE, the professional and the participant were connected by a video call. Following the professional instructions, the participant placed and prepared the necessary material, and after performing the test, the participants registered the results. During the tests, the professional indicated to the participant to position the camera in front of him or her at hip level and far enough away to be able to see the participant completely.

### 2.3. Demographic Information and Quality of Life

Information regarding age, sex, body mass, and height parameters was collected. The Body Mass Index (BMI) calculation (kg/m^2^) was conducted through bodyweight divided by squared height [BMI = weight (kg)/height (m^2^)]. Furthermore, the physical and psychosocial states were registered using the EuroQol-5D (EQ-5D-3L) questionnaire. The questionnaire was composed of 5 dimensions concerning mobility, self-care, activities of daily life, pain, and anxiety/depression [33]. Participants had three levels of scoring options ranging from 1 to 3, being option 1 “I have no problems doing my usual activities” and option three “I am unable to do my usual activities”. In addition, the questionnaire included a Visual Analogue Scale (VAS) in order to self-report the current health state. The VAS is rated from 0 to 100, where higher scores reflect a better health state [33]. The Spanish EQ-5D (3L) version was used [34]. This questionnaire has been employed before to assess the quality of life of chronic back pain patients [7].

#### 2.3.1. The 30 s Chair Stand-Up Test

The participant started the test sitting in the middle of a chair approximately 43 cm from the floor, with the back or legs of the chair against the wall to avoid possible movements [13]. The test began with the participant standing with his or her back straight, feet flat on the floor, and arms crossed over the chest. In the IPE, at the signal “go”, the participant had to stand up and sit down from the chair as many times as possible for 30 s [13]. In the OE, the participants, with a stopwatch, started the test at second 10 and finished it at second 40. The final score was the number of times the participant managed to stand up. The test aimed to assess the strength-endurance of the lower limbs. The performance was measured equally in both test modalities. In the IPE, the professional was the one who counted the repetitions, whereas in the OE, the participant was the one who did it.

#### 2.3.2. Arm Curl Test

The participant began seated in the same chair as in the previous test, without armrests, with their back straight, feet flat on the floor, and the body part of the dominant arm with which he/she performed the test at the end of the chair [13]. The weight selected for the test was 2.300 kg for women and 3.600 kg for men [13]. For this purpose, in both evaluation modalities, the participants added contents to a sturdy cloth or plastic bag until the required weight was reached (e.g., packets of rice, nuts, yogurt, etc.). Although participants were well instructed to place the number of packages needed to reach the weight set in the arm curl test evaluation, it is possible that there may have been slight variations in the total weight incorporated. In the IPE, the test started with the participant holding the weight with his dominant hand and the elbow in extension. To complete the exercise, the participant should flex the elbow and return to full extension of the elbow as many times as possible within 30 s. In the OE, the participants started in the same position, but, with a stopwatch, they started the test at second 10 and finished it at second 40. The performance was measured equally in both test modalities. In the IPE, the professional noted the repetitions performed by the participants, while in the OE the participants counted their own repetitions. The objective was to assess upper limb strength.

#### 2.3.3. 2 Min Step Test in Place

At the signal “go”, the participant started to walk in place by raising the knees until the knees reached the midpoint between the knee and the iliac crest [13]. The number of times the participant raised the knee of the dominant leg for 2 min was recorded [13]. The objective was to assess aerobic endurance. The test was carried out equally in both evaluation modalities, although in the IPE, the professional reported participants’ repetitions, whereas in the OE, the participants counted their own repetitions. Moreover, the 2 min was controlled by the professional in the IPE and by the participant in the OE.

#### 2.3.4. Chair-Sit and Reach Test

The participant was seated on the edge of the chair used in the previous tests and stretched the preferred leg, and the other leg was kept flexed in front of the hip. In the OE, he/she grasped the end of a light, rigid object, e.g., a pen or a ruler, and held it with both hands, fingers outstretched and palms facing each other. The arms remained outstretched with hands together. The participant elongated the trunk by bending the hips with the intention of touching the toe of the outstretched leg. Participants were instructed to stretch the leg parallel to the flexed leg to avoid any hip abduction movements that could interfere with the correct assessment of truck flexibility. In this position, the participant stretched out his arms to their full length holding the object with its major axis parallel to the leg until the end of the object touched the toe. Once this was achieved, the participant simultaneously slid their hands in the object as close to the foot as possible. After stretching as much as possible, the participant was instructed to keep one hand at the maximum point reached. Then, with the other hand, using a ruler or a meter, each participant measured the distance between the end of the object and the fingertips of the hand. In contrast, in the IPE, the original test of the Senior Fitness Test battery was followed [13], so that the participant performed the same procedure as in the OE but without holding an object and keeping his or her hands together and at the same level during the whole test [13]. Therefore, the professional measured the distance reached between the fingertips.

The scoring of both modalities followed the same instructions. If they passed with the fingers, the score had a positive sign (+X cm), if the fingers touched the tip of the toe the scoring was 0 cm, and if they did not reach it, a negative sign before the number was added (−X cm). The objective was to assess the flexibility of the lower body.

#### 2.3.5. Back Scratch Test

In both types of evaluations, the participant was standing with the hand of his/her dominant arm on the same shoulder intending to touch his/her back, from above. In the OE, the dominant hand, outstretched, held an object (e.g., a pen), while the other arm was placed behind the back around the waist with the palm of the hand pointing outwards (in line with the object of the dominant hand). In this way, the end of the object tried to touch the tip of the middle finger of the down hand by adjusting the length of the object left free from the upper hand. As in the previous test, the professional indicated to the participants to keep one hand at the maximum point reached in the object and measured the distance from the tip of the finger of the upper hand and the end of the object. Unlike that, in the IPE, the upper hand of the patient kept its fingers directed downwards and completely stretched trying to reach the fingertip of the other hand, without employing an object [13]. In this case, the professional measured the distance between the tips of the middle fingers. The objective was to assess upper limb flexibility.

The scoring of both modalities followed the same instructions. If they passed, the score had a positive sign (+X cm), if the fingertips of both hands touched each other, the scoring was 0 cm, and if they did not reach it, a negative sign before the number was added (−X cm). The objective was to assess the flexibility of the upper body.

#### 2.3.6. Eight-Foot Up-and-Go Test

Before starting, a chair was placed against the wall, and a cone (or visible mark) was set at a distance of about 2.44 m in a straight line from the back of the chair [13]. At the signal “go”, the participant got up from the chair, without leaning on it, walked to the cone, went around it, and sat back in the chair [13]. The time elapsed between the “go” signal and the moment when the participant sat down on the chair was recorded utilizing a stopwatch. In the IPE, the professional was responsible for controlling the stopwatch, whereas in the OE, the participants started and stopped their stopwatch. In this modality, they were instructed to hold the stopwatch during the test to press the buttons as fast as possible. The aim was to assess agility and dynamic balance.

#### 2.3.7. Sharpened Romberg Test

The test was carried out in a tandem position [30,31]. The participant placed the heel of one foot in contact with the toe of his or her other foot. The time reached in this position was recorded, the test ended when the participant lost his/her balance or when 60 s on the position was reached. In the IPE, the professional started and stopped the stopwatch when the balance was lost or at 60 s, and in the OE modality, the patient controlled it by holding the stopwatch [30,31].

#### 2.3.8. One-Legged Stance Test

The participant made the test with the dominant leg and stood with hands on hips. At the signal “go”, he/she lifted their feet off the ground and placed this leg with the knee flexed in front of him/her. They had to keep their balance for as long as possible. The time that he/she held on without touching the ground with the knee flexed was recorded. The maximum time of the test was 60 s. In the IPE, the professional was responsible for controlling the stopwatch, whereas in the OE modality, the participant started and stropped their own stopwatch by holding it in the hand. The aim was to assess static balance [29,32]. This balance test was proposed since challenging balance task are more sensitive in detecting balance impairments in people with LBP [16,17] than easier ones.

### 2.4. Statistical Analytics

The Statistical Package for Social Science (version 25.0; SPSS, Inc., IBM Corp, Armonk, New York, NY, USA) was used to conduct the statistical analyses. Data are presented as the mean and standard deviation (SD) from each evaluation modality and from the difference between modality mean results. The explained statistical analysis procedures were performed based on the global sample size, as well as by dividing the participants according to their sex. The alpha level of significance was set at <0.05.

Furthermore, parametric and non-parametric tests were conducted based on the Shapiro–Wilk results. Validity is a multicomponent concept that assesses to what extent a tool measures what it is intended to measure [35]. Criterion validity is an estimate of the extent to which a measure agrees with a gold standard, and it is possible to evaluate by statistically testing a new measurement technique against an independent criterion or standard (concurrent validity) [36]. For this reason, to explore the concurrent validity, the performance differences between the evaluation modalities (IPE and OE), the one sample *t*-test or one-sample Wilcoxon signed rank test was used. In the same vein, the intraclass correlation coefficient (ICC) of two-way mixed effects, consistency, and single rater/measurement [37] were calculated to assess the level of agreement between OE and the IPE results. ICC values less than 0.50 are indicative of poor agreement, values between 0.5 and 0.75 indicate moderate agreement, values between 0.75 and 0.90 indicate good agreement, and values greater than 0.90 indicate excellent agreement [38]. Moreover, Bland-Altman plots were performed to illustrate the agreement between the OE and IPE scores [39] to provide the limits of agreement and bias, following the procedure proposed by Giavarina [40]. The software Graphpad Prism 8.0 was used to create the graphs. Lastly, the correlations between the tests of the two measurement methods were analyzed, and Cohen’s recommendations [41] were followed to interpret the correlation coefficient. A score ≥0.5 was strong, it was moderate if the score was between 0.5 and 0.35, and it was poor if the score was ≤0.35.

## 3. Results

A total of 26 women and 14 men (65% and 35% of the total sample size, respectively) participated in this study. Their age ranged from 45 to 72 years. Regarding BMI results, 53.84% of the women had a normal weight, 38.46% were overweight, and 7.69% were obese, whereas 35.71% of the men had a normal weight, 50% were overweight, and 14.28% were obese.

Participants’ quality of life (EQ-5D-3L coefficient) reached high scorings close to 1 (full healthy), the maximum achievable score. Concerning the EQ-5D Visual Analogue Scale, participants also perceived their health to be apparently elevated since it was close to 100. Analyzing the included dimensions, the highest quality of life problem was obtained in pain perception. More specific information about participants’ characteristics is shown in Table 2.

**Figure 2:**
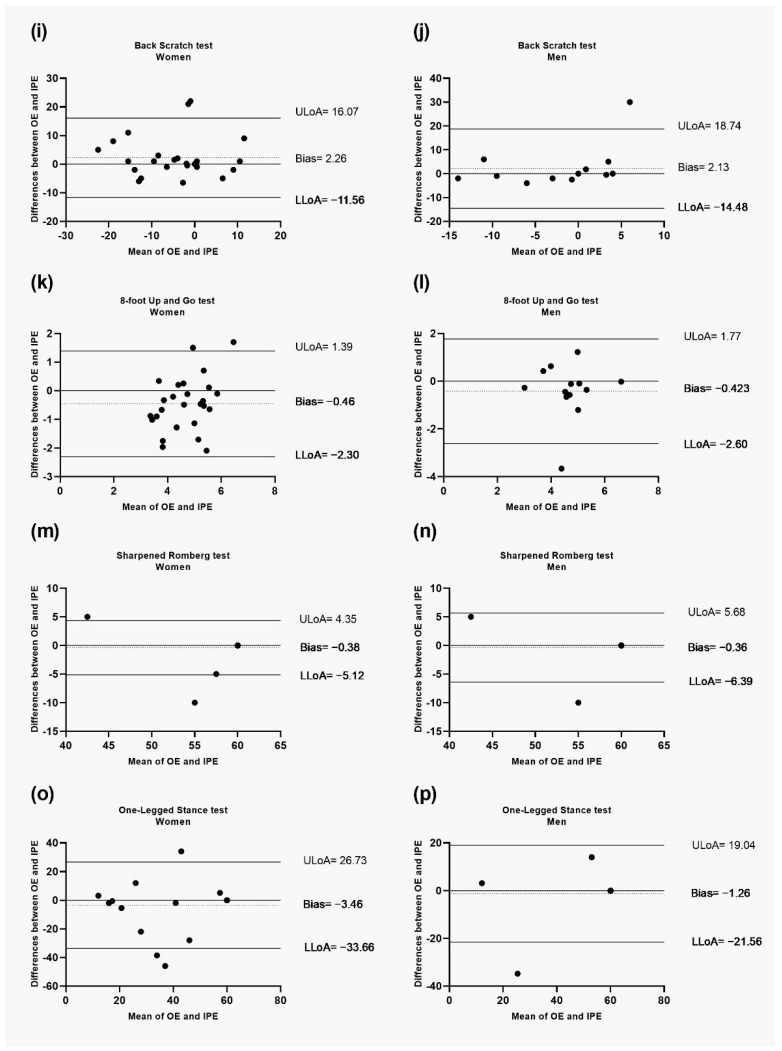
Bland-Altman plots for the difference between online evaluation and face-to-face evaluation of the SFT and the two balance test for participants divided by sex. (**i**) Back Scratch test for women; (**j**) Back Scratch test for men; (**k**) 8-foot Up and Go test for women; (**l**) 8-foot Up and Go test for men; (**m**) Sharpened Romberg test for women; (**n**) Sharpened Romberg test for men; (**o**) One-Legged Stance test for women; and (**p**) One-Legged Stance test for men. OE: online evaluation; IPE: face-to-face evaluation; ULoA: upper limit of agreement; LLoA: lower limit of agreement. The shaded areas represent the confidence interval limits for mean and agreement limits.

Table 3 shows participants´ descriptive data and the agreement in the OE and IPE of the total sample size. Comparisons between OE and IPE did not show significant differences between OE and IPE modalities in all tests (*p* > 0.05), except in the 8-foot up-and-go (*p*-value = 0.007). ICC values ranged from moderate to excellent agreement regarding the lowest and highest score (0.55 to 0.93). A moderate agreement was found for the arm-curl test and the 8-foot up-and-go test. In addition, a good agreement was found for the 30 s chair stand-up test, 2 min step-test in place, back scratch test, sharpened Romberg test, and the one-legged stance test. Moreover, excellent agreement was found in the chair-sit and reach test. Considering the Bland-Altman plots of the scores obtained by OE and IPE of the tests of the total sample size (see Figure 2), the graphs depict excellent agreement between OE and IPE with only reduced scores of observations falling outside the limits of agreement for the different test performed. Moreover, there was not significant bias since the line of equality was within the confidence interval of the mean difference. Finally, all variables showed strong correlations, except for the arm curl test and 8-foot up-and-go test, where moderate correlations were found. However, all variables showed significant relationships.

Agreement data divided by sex is reported in the Supplementary Materials (See Supplementary Table S1).

Figure 2 shows the Bland-Altman plots of the scores obtained by OE and IPE of the tests of the total sample size. Moreover, in the Supplementary Materials, the Bland-Altman plots for women and men in the OE and IPE are reported (See Supplementary Figures S1 and S2). The plots of the total sample size (Figure 2) and the men and women analysis (Supplementary Figures S1 and S2) depicted excellent agreement between the OE and IPE with only reduced scores of observations falling outside of the limits of agreement for the different tests performed.

## 4. Discussion

The Senior Fitness test battery [13] consists of six physical fitness tests that assess strength, flexibility, cardiorespiratory fitness, and agility (30 s chair stand-up, arm curl, 2 min step, chair-sit and reach, back scratch test and 8-foot up-and-go). This battery is a useful tool to evaluate people with LBP since the ACSM recommends the evaluation of these physical capacities, concretely cardiorespiratory fitness, strength, and flexibility [12]. In addition, previous studies have shown balance impairment in people with LBP [14,15]. Thus, the validity of an OE in contrast to IPE through the level of agreement in different functionality tests was investigated. The results achieved with the present investigation showed reasonable levels of agreement regarding the ICC values [38], the Bland-Altman plots between the variables of the different evaluation methods [39], and the strong correlations obtained in all tests except for the arm curl test and the 8-foot up-and-go test, where moderate correlations were found. Nevertheless, all variables showed significant relationships between OE and IPE. Therefore, the initial hypothesis proposed should be accepted. These results may prove to be of great relevance in the clinical field in order to have objective tools to face current practical challenges.

With the COVID-19 pandemic [19], the growth of telemedicine, and the emergence of new technologies and Internet access, physical fitness assessments have considered new ways to monitor and control the functionality and physical condition of participants. The validation of online functional tests in comparison to the gold standard face-to-face test, as performed in the present study, can be of great help in clinical rehabilitation units, especially when hospitals are overcrowded or when, for health reasons, patients cannot be assessed face-to-face [42]. In this way, rehabilitation staff can remotely assess the physical function of patients without direct contact, and it would be possible to assess patients more objectively and quickly prior to their incorporation into rehabilitation or training programs. This clinical implication, that the incorporation of online testing in patients with LBP could foster the autonomy and self-determination of those suffering from LBP, is necessary in the effectiveness of their care [43].

Physical exercise home-based interventions have emerged as an effective way to maintain physical and mental health [44,45]. However, these home-based physical exercise interventions should monitor and control the participants’ physical fitness. In this regard, previous studies have reviewed [21] and investigated the reliability of different physical fitness tests using remote evaluation [24]. Ogawa, Harris, Dufour, Morey, and Bean [24] showed high inter-rater reliability in three tests used in the present study, the 30 s arm curl, 30 s chair stand test, and 2 min step test, among community-dwelling older adults. However, remote physical fitness assessment was not compared and validated with a face-to-face evaluation. In the same line, Lin et al. [46], showed moderate-to-good reliability of the fitness assessments using a health app. Again, this remote evaluation was not compared with a face-to-face condition.

Nevertheless, some studies compared face-to-face evaluations with remote evaluations in populations different from people with LBP. Holland, et al. [47] examined the usability of the 6 min walking test at home. Results showed that this test underestimated the exercise capacity when conducted at home in people with chronic obstructive pulmonary disease. Similarly, Cox et al. [48] showed that exercise capacity assessment, using the 3 min step test, is feasible and accurate via remote videoconferencing in adults with cystic fibrosis. However, the OE conducted in these studies has not been compared to the IPE evaluation. In this regard, Winters-Stone, Lipps, Guidarelli, and Herrera-Fuentes [25] reported the validity of the chair stand test, showing a good correlation between an EO and IPE in older adults with cancer (r = 0.81). The results achieved with the present study showed a lower correlation value (correlation coefficient = 0.66) in people with LBP. Hoenemeyer, Cole, Oster, Pekmezi, Pye, and Demark-Wahnefried [26] reported the reliability and validity of the SFT (including the sit and reach test, the 30 s chair stand test, the back scratch test, 8-foot up-and-go test and go test, the timed 8 min walk test, and the 2 min step test) in cancer survivors and supportive partners. In this previous study, the authors reported the validity through the ICC, showing the highest value in the back scratch test (ICC = 0.95), classified as excellent. The other tests (30-s chair stand-up, 2 min step, chair-sit and reach, 8 min walk test, and 8-foot up-and-go) also showed high validity values. The results presented showed a lower ICC value (ICC = 0.79) in the back scratch, although validity can be considered good. In addition, they showed similar ICC values in the chair-sit and reach (ICC = 0.87) and the 2 min step (ICC = 0.93) compared to those in the Hoenemeyer, Cole, Oster, Pekmezi, Pye, and Demark-Wahnefried [26] study (ICC = 0.89 and ICC = 0.84 for the sit and reach and 2 min step test, respectively).

The greatest differences in validity were observed in the 8-foot up-and-go test with an ICC = 0.64 compared to an ICC = 0.80 observed in a previous study [26]. This could be due to the difference in the test performance, being higher in the present study than in the study focused on cancer survivors [26]. Furthermore, another possible explanation for this result can be extracted. In this regard, the lowest ICC values were obtained by the arm-curl test (ICC = 0.55) and the 8-foot up-and-go test (ICC = 0.64). In the same line, these two tests are those that yielded the lowest correlation values (arm-curl test correlation coefficient = 0.38 and the 8-foot up-and-go test correlation coefficient = 0.48). Regarding the arm-curl test, participants in the OE condition were asked to start the test when their stopwatch was at second 10 and finished it at second 40. Since participants would have paid attention to the stopwatch, the participants would be less accurate in measuring their performance on this test. Similarly, in the 8-foot up-and-go test, the stopwatch was controlled by the participant themself in the OE condition, whereas in the IPE condition, the stopwatch was controlled by the research staff. This would make the time the participant took to hit play and stop longer. Future studies should take this issue into account.

This study has some limitations that should be acknowledged. First, the investigation conducted was observational and monocentric with a sample composed of people with LBP, so results cannot be generalized to other populations. In addition, the sample of men was relatively small. Second, a test-retest analysis was not conducted. Thus, future studies should analyze the reliability of the physical fitness battery for OE and IPE. Third, it would have been interesting to specify the camera angle and the distance at which the device was located to facilitate the replication of the conditions of this study, although this is a difficult task since the conditions of each device vary according to the manufacturer. Fourth, it is possible that in the arm curl test, there were slight variations in the total weight incorporated into the sturdy cloth or plastic bag. In the same vein, it is also possible that in the back scratch test, the chair sit and reach test, and the 8-foot up-and-go test, there were small alterations due to the difficulty in performing the test or the time invested in pressing the stopwatch, respectively. Considering these limitations, results can be taken with caution. Although this study has some limitations, strengths should also be highlighted. In this regard, to the best of our knowledge, this is the first study that analyzed the validity between OE and IPE in an LBP population. Furthermore, unlike previous studies, the present investigation included a wide range of physical fitness tests: strength, flexibility, agility, cardiorespiratory fitness, and balance. In addition, the present results provide relevant information to clinical and physical specialists who want to implement online or home-based physical exercise interventions. In this regard, the physical fitness tests have been modified so that patients can be self-assessed. This would allow healthcare professionals and patients to economize time and aid resources while maintaining validity.

## 5. Conclusions

The results achieved in the current investigation showed reasonable levels of agreement that support the validity between OE and IPE in all tests regarding the ICC values, the Bland-Altman plots between the variables of the different evaluation methods, and the strong correlations obtained, except for the arm curl test and the 8-foot up-and-go test, where moderate correlations and lower ICC values were found. However, it is important to take into account the range of fluctuation of the ICC and the significant values obtained through correlations.

## Figures and Tables

**Table 1:** Physical and measurement adaptations in the face-to-face and online evaluations for test self-administration.

Original Measure	Adaptions for the Face-to-Face Evaluation	Adaptions for the Online Evaluation
30 s Chair Stand-Up Test [13]	Same as original	Participants controlled the stopwatch by starting the test at second 10 and finishing it at second 40. Repetitions were counted by participants.
Arm Curl Test [13]	The weight was added to a sturdy cloth or plastic bag until 2.300 kg and 3.600 kg was reached (e.g., packets of rice, nuts, yogurt, etc.).	
		Participants controlled the stopwatch by starting the test at second 10 and finishing it at second 40. Repetitions were counted by participants.
2-min Step Test in Place [13]	Same as original	Repetitions were counted by participants. Time was controlled by each participant.
Chair-sit and Reach Test [13]	Same as original	A light object, such as a ruler, was held with both hands by the participants. Participants positioned the end of the object touching the toe tips and slid their hands elongating the trunk. The score was obtained by measuring the distance between the fingertip and the end of the object.
Back Scratch Test [13]	Same as original	A light object, such as a ruler, was held with the upper hand until the end touched the fingertip of the lower hand. The upper hand slid in the object as low as possible. The score was obtained by measuring the distance between the fingertip and the end of the object.
8-Foot Up-and-go Test [13]	Same as original	Participants started and stopped their stopwatch holding it during the test.
Sharpened Romberg Test [31]	Same as original	Participants controlled the stopwatch by pressing start and stop finishing the test when 60 s was reached or balance was lost.
One-Legged Stance Test [32]	Same as original	Participants controlled the stopwatch by pressing start and stop finishing the test when 60 s was reached or balance was lost.

**Table 2:** Participants’ physical and quality-of-life characteristics.

Participants’ Characteristics	Total Sample Size	Women (*n* = 26)	Men (*n* = 14)
	Mean (SD) [range of variability]	Mean (SD) [range of variability]	Mean (SD) [range of variability]
Age	58.48 (9.87) [45–72]	58.46 (9.23) [45–72]	58.50 (10.14) [47–69]
Height (cm)	166.00 (9.72) [145–184]	164.38 (10.27) [145–176]	169.00 (8.09) [165–184]
Body mass (Kg)	71.10 (13.08) [53–107]	69.92 (14.52) [53–88]	73.29 (10.01) [65–107]
BMI	25.69 (3.37) [18.78–35.34]	25.74 (3.82) [18.78–31.55]	25.59 (2.42) [21.50–35.34]
EuroQol-5D (coefficient)	0.85 (0.14) [0.32–1]	0.87 (0.12) [0.74–1]	0.81 (0.18) [0.32–1]
EuroQol-5D (VAS)	76.25 (10.49) [60–100]	76.73 (11.13) [60–100]	69.87 (9.50) [60–90]
Mobility	1.08 (0.27) [1–2]	0.87 (0.12) [1–2]	1.21 (0.43) [1–2]
Self-Care	1.03 (0.16) [1–2]	1.04 (0.20) [1–2]	1.00 (0.00) [1]
Activities Of Daily Life	1.20 (0.41) [1–2]	1.19 (0.40) [1–2]	1.21 (0.43) [1–2]
Pain	1.63 (0.54) [1–2]	1.62 (0.50) [1–2]	1.64 (0.63) [1–2]
Anxiety/Depression	1.05 (0.22) [1–2]	1.00 (0.00) [1]	1.14 (0.36) [1–2]

SD: standard deviation, EuroQol-5D: European Quality of Life-5 Dimensions, VAS: visual analogue scale.

**Table 3:** Agreement under online and face-to-face evaluations of total sample size (N = 40).

Variable	Online Evaluation Mean (SD)	Face-to-Face Evaluation Mean (SD)	*p*-Value	ICC (95% CI)	Correlation Coefficient
30 s Chair Stand-Up test	19.00 (3.76)	18.18 (3.91)	0.108	0.79 (0.610–0.891)	0.66 ***
Arm-curl test	21.38 (4.42)	20.70 (4.28)	0.385	0.55 (0.143–0.760)	0.38 *
2 min step-test in place	107.50 (23.12)	110.10 (23.13)	0.306	0.87 (0.748–0.930)	0.76 ***
Chair-sit and reach test	−1.46 (9.59)	−0.93 (10.50)	0.528	0.93 (0.859–0.961)	0.86 ***
Back scratch test	−4.89 (8.53)	−2.68 (9.25)	0.305	0.79 (0.596–0.887)	0.64 ***
8-foot up-and-go test	4.89 (0.85)	4.45 (1.06)	0.007	0.64 (0.313–0.808)	0.48 **
Sharpened Romberg test	59.00 (4.41)	58.63 (3.92)	0.334	0.89 (0.792–0.942)	0.66 ^†††^
One-legged stance test	50.57 (16.34)	47.88 (19.44)	0.363	0.83 (0.675–0.909)	0.75 ^†††^

SD: standard deviation, ICC: intraclass correlation coefficient. Correlation Coefficient. Paired *t*-test or Wilcoxon tests were conducted depending on the distribution (variables with a *p*-value lower than 0.05 in the Shapiro–Wilk test were considered for a non-parametric analysis). *** *p*-value < 0.001; ** *p*-value < 0.01; * *p*-value < 0.05 based on Pearson’s correlation coefficient. ^†††^ *p*-value < 0.001; based on Spearman’s Rho correlation coefficient.

## Data Availability

Data will be available upon reasonable request to the corresponding author.

## Supplementary Materials

The following supporting information can be downloaded at: https://www.mdpi.com/article/10.3390/healthcare11071019/s1, Supplementary Table S1. Agreement under online and face-to-face evaluations divided by women (*n* = 26) and men (*n* = 14). Supplementary Figure S1. Bland-Altman plots of the SFT and the two balance tests for participants divided by sex. OE: online evaluation; IPE: face-to-face evaluation; ULoA: upper limit of agreement; LLoA: lower limit of agreement. The shaded areas represent the confidence interval limits for the mean and agreement limits. Supplementary Figure S2. Bland-Altman plots of the SFT and the two balance tests for participants divided by sex. OE: online evaluation; IPE: face-to-face evaluation; ULoA: upper limit of agreement; LLoA: lower limit of agreement. The shaded areas represent the confidence interval limits for the mean and agreement limits.
