# Supplementary material for: Validity of On-Line Supervised Fitness Tests in People with Low Back Pain
Source: Healthcare (Basel). 2023 Apr 3;11(7):1019. doi: 10.3390/healthcare11071019 (PMC10094436; doi:10.3390/healthcare11071019)
Supplement: Supplementary file 1 [file healthcare-11-01019-s001.zip › healthcare-2263380-supplementary.pdf]

Supplementary Material

Supplementary Table S1 shows the agreement parameters obtained between IPE and OE scores divided by women and men. For women, comparisons between OE and IPE did not show significant differences between OE and IPE modalities in all the test (p>0.05) except in the 30s chair stand-up test (p-value= 0.043) and the 8-foot up and go (p–value= 0.021). ICC values ranged from poor to excellent agreement regarding the lowest and the highest score (0.40 to 0.94). First, a poor agreement was found for the arm-curl test. Second, moderate agreement was found for the 30s chair stand-up test and the 8-foot up-and-go test. Third, a good agreement was found for, the 2 min step-test in place, back scratch test, sharpened Romberg test and the one-legged stance test. Finally, an excellent agreement was found for the chair-sit and reach. All variables showed strong correlations except for the arm curl test where poor correlation was found. However, all variables showed significant relationships.

For men, comparisons between OE and IPE only showed significant differences between OE and IPE modalities in the 2 min step-test in place (p-value= 0.012). ICC values ranged from moderate to excellent regarding the lowest and the highest score (0.54 to 0.92). In the first place, a moderate agreement was found for the arm curl test, back scratch test and the 8-foot up-and-go test. Second, a good agreement was found for the 30s chair stand-up test and the one-legged stance test. Finally, an excellent agreement was found for the 2 min step-test in place, chair-sit and reach test and the sharpened Romberg test. Considering the Bland-Altman plots of the scores obtained by OE and IPE of the tests of the sample size divided by sex the graphs depict excellent agreement between OE and IPE with only reduced score of observations falling outside the limits of agreement for the different tests performed. Moreover, there is not significant bias since the line of equality is within the confidence interval of the mean difference. All variables showed strong correlations except for the arm curl test and back scratch test where moderate correlations were found, and the 8-foot Up and Go test where poor correlation was found. However, all variables showed significant relationships.

Supplementary Table S1. Agreement under online and face-to-face evaluations divided by women (n=26) and men (n=14).

Variable		Online evaluation Mean (SD)	Face-to-face evaluation Mean (SD)	*p*-value	ICC (95% CI)	Correlation Coefficient
30s Chair Stand-Up test	W	18.03 (3.34)	16.77 (3.34)	0.043	0.74 (0.417-0.883)	0.59**
	M	20.79 (3.95)	20.79 (3.62)	1.000	0.76 (0.241-0.922)	0.61*
Arm-curl test	W	20.88 (4.85)	19.88 (4.20)	0.342	0.49 (0.000-0.773)	0.33
	M	22.29 (3.45)	22.21 (4.15)	0.949	0.59 (0.000-0.868)	0.43
2 min Step-test in place	W	107.50 (22.74)	106.46 (24.47)	0.753	0.86 (0.683-0.936)	0.75**
	M	107.50 (24.69)	116.86 (19.46)	0.012	0.92 (0.752-0.974)	0.88**
Chair-sit and Reach test	W	0.17 (9.40)	-0.38 (8.81)	0.508	0.94 (0.873-0.974)	0.89**
	M	-4.49 (9.52)	-1.93 (13.41)	0.167	0.91 (0.732-0.972)	0.89**
Back Scratch test	W	-5.56 (9.66)	-3.30 (9.51)	0.218	0.84 (0.651-0.930)	0.73**
	M	-3.64 (6.02)	-1.51 (8.97)	0.929	0.56 (0.000-0.857)	0.42
8-foot Up and Go test	W	4.90 (0.80)	4.45 (1.10)	0.021	0.69 (0.297-0.859)	0.55**
	M	4.86 (0.98)	4.45 (1.01)	0.185	0.54 (0.000-0.853)	0.37
Sharpened Romberg test	W	59.23 (3.92)	58.85 (3.55)	0.414	0.88 (0.740-0.948)	0.60††
	M	58.57 (5.35)	58.21 (4.64)	0.655	0.90 (0.675-0.966)	0.73†
One-Legged Stance test	W	48.59 (17.50)	45.13 (20.02)	0.308	0.80 (0.550-0.910)	0.70††
	M	54.24 (13.80)	52.98 (17.89)	1.000	0.88 (0.634-0.962)	0.84††

Abbreviations: W; Women, M; Men, SD; Standard Deviation, ICC; Intraclass Correlation Coefficient. Paired *t*-test or Wilcoxon tests were conducted depending on the distribution (variables with a *p*-value lower than 0.05 in the Shapiro–Wilk test were considered for a non-parametric analysis). ***p*-value<0.001; **p*-value<0.05 based on Pearson's correlation coefficient. ††*p*-value<0.001; †*p*-value<0.05 based on Spearman's Rho correlation coefficient.

Supplementary Figure S1 and Supplementary Figure S2 show the Bland-Altman plots of the scores obtained in the OE and IPE for women and men.

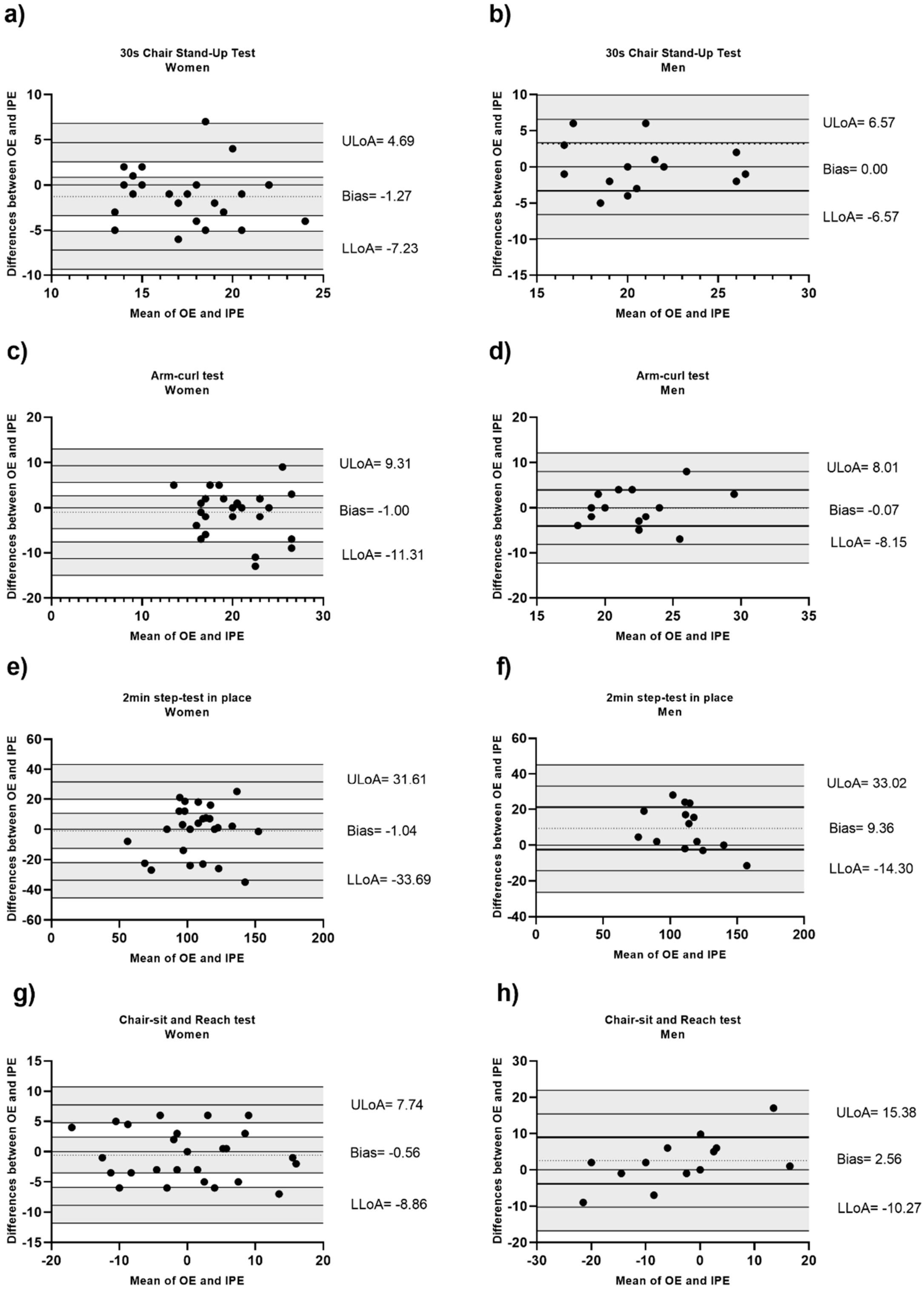

Supplementary Figure S1. Bland-Altman plots of the SFT and the two balance tests for participants divided by sex. OE: Online evaluation; IPE: Face-to-face evaluation; ULoA: Upper limit of agreement; LLoA: Lower limit of agreement. The shaded areas represent the confidence interval limits for mean and agreement limits.

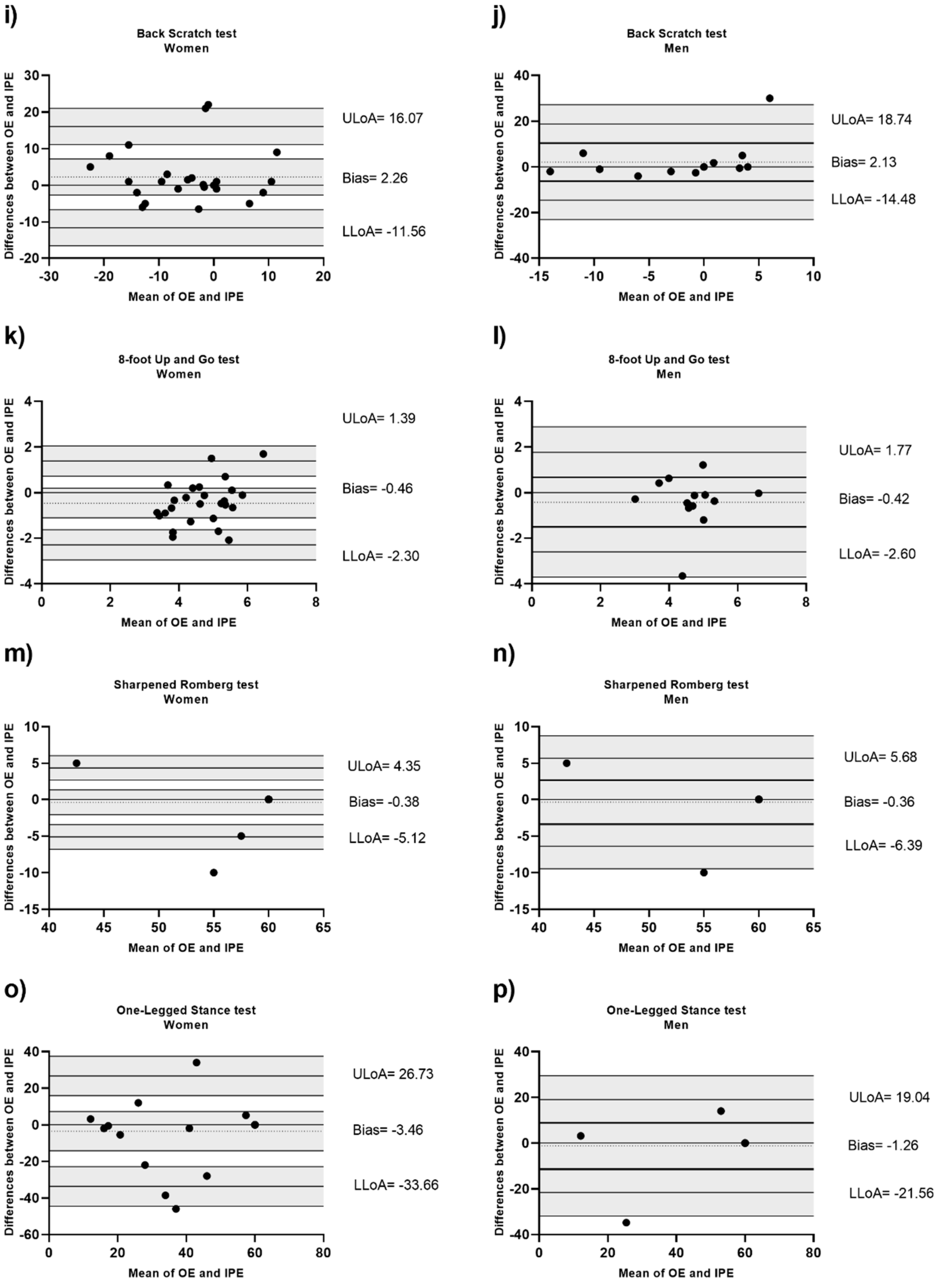

Supplementary Figure S2. Bland-Altman plots of the SFT and the two balance tests for participants divided by sex. OE: Online evaluation; IPE: Face-to-face evaluation; ULoA: Upper limit of agreement; LLoA: Lower limit of agreement. The shaded areas represent the confidence interval limits for mean and agreement limits.
